# Silica Monolith for the Removal of Pollutants from Gas and Aqueous Phases

**DOI:** 10.3390/molecules26051316

**Published:** 2021-03-01

**Authors:** Vanessa Miglio, Chiara Zaccone, Chiara Vittoni, Ilaria Braschi, Enrico Buscaroli, Giovanni Golemme, Leonardo Marchese, Chiara Bisio

**Affiliations:** 1 Department of Sciences and Technological Innovation and Interdisciplinary Nano-SiSTeMI Centre, University of Eastern Piedmont A. Avogadro, Viale T. Michel 11, 15121 Alessandria, Italy; vanessa.miglio@uniupo.it (V.M.); chiara.zaccone@uniupo.it (C.Z.); chiara.vittoni@uniupo.it (C.V.); leonardo.marchese@uniupo.it (L.M.); 2 Interdisciplinary Nano-SiSTeMI Centre, University of Eastern Piedmont A. Avogadro, Viale T. Michel 11, 15121 Alessandria, Italy; 3 Department of Agricultural and Food Sciences, University of Bologna, Viale G. Fanin 44, 40127 Bologna, Italy; enrico.buscaroli2@unibo.it; 4 Department of Environmental Engineering, University of Calabria, Via P. Bucci 45A, 87036 Rende, Italy; giovanni.golemme@unical.it; 5 CNR-SCITEC Istituto di Scienze e Tecnologie Chimiche “Giulio Natta”, Via G. Venezian 21, 20133 Milano, Italy

**Keywords:** adsorption, toluene, rhodamine B, water stability of monolith

## Abstract

This study focused on the application of mesoporous silica monoliths for the removal of organic pollutants. The physico-chemical textural and surface properties of the monoliths were investigated. The homogeneity of the textural properties along the entire length of the monoliths was assessed, as well as the reproducibility of the synthesis method. The adsorption properties of the monoliths for gaseous toluene, as a model of Volatile Organic Compounds (VOCs), were evaluated and compared to those of a reference meso-structured silica powder (MCM-41) of commercial origin. Silica monoliths adsorbed comparable amounts of toluene with respect to MCM-41, with better performances at low pressure. Finally, considering their potential application in water phase, the adsorption properties of monoliths toward Rhodamine B, selected as a model molecule of water soluble pollutants, were studied together with their stability in water. After 24 h of contact, the silica monoliths were able to adsorb up to the 70% of 1.5 × 10^−2^ mM Rhodamine B in water solution.

## 1. Introduction

In recent years, chemical industries have been focusing on sustainable development approaches, promoting products and services that offer performance at lower costs, reducing significantly the environmental impact and improving the quality of life.

In connection to these approaches, mesoporous materials have been synthesized in the form of powder and extensively studied for various applications (i.e., catalysis, adsorption, etc.). Nevertheless, on an industrial level, the use of powders for environmental applications has been severely hindered due to their handling and recycling limitations. Therefore, in recent years, research has been focusing on developing materials on a macroscopic scale, aiming at facilitating their handling, recovery and reuse in order to expand the range of their applications to different fields [1].

To overcome problems related to the use of powders there are two possible options: (i) use pre-synthesized silica powders to form pellets, or (ii) directly synthesize silica monoliths. In the first case, it is often necessary to use one or different binders (such as methylcellulose) and to press the materials even under heating. This multistep time-consuming procedure could adversely affect the structure of mesoporous silica, thus causing performance alteration [2]. In the second case, the formation of silica monolith can be a convenient way to fully exploit the structural and functional properties of the material [3] by saving, at the same time, both reactants and time, in that a single-step synthesis is required.

Several studies have been carried out in the recent literature to obtain monolithic siliceous structures. Galarneau et al. synthetized macroporous silica monoliths with disordered mesoporosity, prepared by the spinodal decomposition method [4]. Nakanishi and coworkers synthesized silica gel monoliths with macro-mesoporous hierarchical structure via a spontaneous sol–gel process from silicon alkoxide using a structure-directing agent and a micellar swelling agent [5]. Kohns et al. obtained silica monoliths with submicrometric macropores, introducing urea as an agent to control the size of macropores, mesopores and skeleton thickness [6]. Fotoohi et al. prepared mesoporous silica monoliths using a simple one-pot sol–gel synthesis with subsequent atmospheric evaporation [1]. Roucher et al. synthesized self-supported macro-mesoporous SBA-15-Si (HIPE) monoliths by combining emulsion and cooperative templating mechanisms [7].

Silica monoliths have been used in applications mainly related to the field of chromatography for the preparation of columns [8]. However, recent examples report on their use for the removal of chemical compounds such as fatty acids, phenols, and sterols from wastewater effluents: in these cases, they are mainly exploited as composite [9] or biocomposite materials [10] after the inclusion of biomolecules or enzymes. Few examples of the use of silica monolith also concern the preparation of composite [5] or functionalized materials for CO_2_ adsorption [4,11]. Organic-modified silica monoliths are also used for the sequestration of heavy metals and for the immobilization of enzymes [12,13].

VOCs (Volatile Organic Compounds) are a group of noxious organic compounds characterized by vapor pressure higher than 0.01 kPa at 293.15 K. The group includes some of the most common air pollutants released by chemical, petrochemical and allied industries [14] such as aliphatic, aromatic and chlorinated hydrocarbons, aldehydes, terpenes, alcohols, esters and ketones. Due to their toxicity, the removal of these compounds by means of adsorbent materials [15] is of environmental concern and a relevant issue for human health. Several recent studies focused on the use of mesoporous ordered silicas, functionalized with organic groups, for the adsorption of VOCs [16]. The toluene adsorption properties of different porous silicas (i.e., fumed silica, SBA-15 and commercial MCM-41) have been recently reported by our group [16] and we have shown that the porous architecture of different silicas have an important effect on the final adsorption properties. In the same conditions, we also tested the adsorption behaviour of siliceous zeolites and hypercross-linked polymers [17]. We derived that whereas siliceous zeolite adsorbs 21 Q% of toluene, HyperCross-linked Polymers (HCP_S_) with flexible structures are able to adsorb more than 140 Q% of toluene.

Soluble dyes, such as Rhodamine B, Methylene blue, Congo red, Methyl Orange, etc., have been increasingly used for applications in different fields (i.e., food, paper, textiles, paints, pharmaceuticals, cosmetics, etc.) [18]. Their occurrence in industrial wastewater is worrying because they are harmful for ecosystems as well as animals and humans [19]. Although there are many treatment methods for removing dyes from aqueous solutions (e.g., chemical oxidation, photodegradation, membrane filtration), adsorption has been found to be a high-efficient and low-cost technology [20] and several sorbent materials such as activated carbons [21], clays [22], zeolites [23], activated alumina [24], etc., have been tested for this purpose. Among these, ordered mesoporous silicas are promising candidates due to their high specific surface area, uniform pore size and high pore volume.

For example, Rasalingam et al. showed that mesoporous MCM-48 and MCM-41 are able to remove, respectively, 87% and 81% of Rhodamine B from an aqueous solution at the concentration of 10^−5^ M [25]. They found that, in the used conditions, the MCM-41 sample (having a specific surface area of 1453 m^2^·g^−1^) is able to retain 8.2 × 10^−4^ mol·g^−1^ of Rhodamine B. Others used MCM-41 (specific surface area of 1300.5 m^2^·g^−1^) to remove Rhodamine B at 25 °C and pH = 4 and in these conditions the solids absorb ca. 9.4 × 10^−4^ mol·g^−1^ [26].

In this work, for the first time, mesoporous silica monoliths without any surface functionalization are proposed for the removal of organic pollutants from water and for the reduction of volatile organic compounds (VOCs). Model molecules (i.e., Rhodamine B and toluene, respectively) are used to this purpose. Our aim is to study the adsorption capacities of a silica-based monolith intended as a possible candidate to overcome limitations related to the use of mesoporous silica in powder form (i.e., handling and recycling issues). We focused on the optimization of a synthesis procedure for the direct formation, in a single work phase, of cylindrical mesoporous silica monoliths and to the determination of their physico-chemical properties by using different experimental techniques. The spatial textural homogeneity of the synthesized monoliths and the reproducibility of the synthesis were assessed. Moreover, the monoliths were tested for the removal of toluene, chosen as a model molecule of aromatic hydrocarbons, from gas phase. A combination of FT-IR spectroscopy and volumetric analysis was adopted to study the adsorption process and gain knowledge on the interactions between adsorbent surface and toluene molecule. Finally, the ability of the same monoliths to remove Rhodamine B from aqueous solution was also studied by using UV-Visible spectroscopy. Fundamental parameters of the monoliths for real water applications (i.e., mechanical and textural stability under operating conditions) [27] were verified as well.

## 2. Results and Discussion

### 2.1. Physico-Chemical Characterization of Silica Monoliths

**Figure 1:**
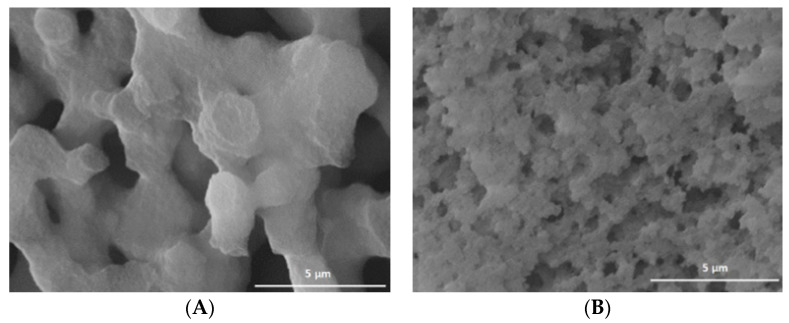
SEM micrographs (20000×) of Mono-ICE before (**A**) and after calcination (**B**).

The morphology of the silica monoliths, named Mono-ICE (see Section 3.1), was studied by using scanning electron microscopy (SEM). The obtained micrographs of samples before (A) and after the calcination step (B) to remove the polyethylene oxide template (PEO, see Section 3.1), are reported in Figure 1.

The Mono-ICE sample after the spinodal phase separation process (Figure 1A) is characterized by an irregular morphology, where the particle aggregation gives origin to interconnected macropores with a diameter of 4 μm and a skeleton thickness of 3 µm, on average, in accordance with what it has been reported in the reference article [4]. After the formation of the macroporous monoliths in acidic medium at low temperature, the ammonia treatment at 40 °C brings about the rearrangement of the weakly condensed silica network into denser nanodomains and the formation of larger mesopores, giving rise to the final hierarchical SBA-15 [4]. After the calcination of the organics (Figure 1B), the sample morphology appears deeply modified, with a strong reduction of macropore dimensions to 0.5–1 µm, on average.

The surface properties of the calcined silica monolith were then monitored by infrared spectroscopy (Figure S1 from Supplementary Materials). The FT-IR spectrum of Mono-ICE sample (Figure S1 from Supplementary Materials) is characterized by the presence of an intense band at 3743 cm^−1^, due to the O-H stretching mode of isolated silanol groups present on the silica surface, and a broad band between 3700 and 3200 cm^−1^ with a maximum at 3521 cm^−1^ and a shoulder at ca. 3654 cm^−1^, due to different surface silanol groups interacting with each other through hydrogen bonds. In the low frequency region, the spectrum shows three bands at 1983, 1863 and 1637 cm^−1^, due to overtones and combination modes of the silica framework [28].

**Figure 2:**
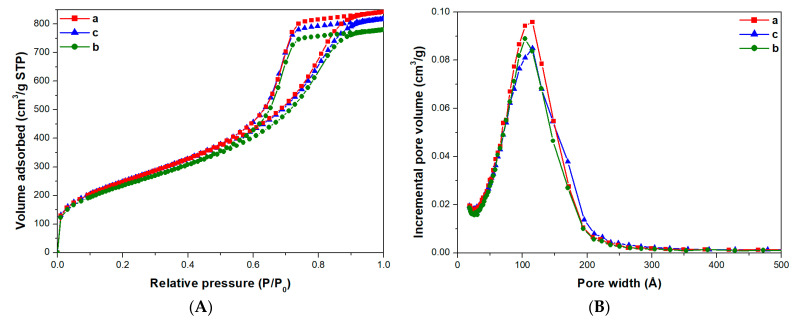
N_2_ adsorption and desorption isotherms at 77K (**A**) and pores size distribution (**B**) of calcined Mono-ICE-Lateral A (a), Mono-ICE-Lateral B (b), and Mono-ICE-Central C (c).

Textural properties of the monoliths were determined by N_2_ adsorption–desorption isotherms at 77 K. In order to investigate whether the physico-chemical properties were homogeneous throughout the entire length of the cylindrical monoliths, several samples were singly divided into three parts of 1 cm in length each and of a weight of about 50 mg: the obtained samples were named Mono-ICE-Lateral A, Mono-ICE-Lateral B, Mono-ICE-Central C, respectively. Nitrogen adsorption and pore size distribution of the samples are shown in Figure 2A,B, respectively.

Following the IUPAC classification, all the isotherms obtained (Figure 2A) are of type IVa, thus indicating a multilayer adsorption, which is typical of mesoporous solids. Hysteresis loops of type H2, due to disordered materials with a distribution of pore size and shape that is not well-defined, are found. All samples show a monomodal pore size distribution between 50 and 200 Å with maxima at around 110 Å (Figure 2B). The textural features of the three samples are similar, and this strongly suggests that the cylindrical monolith under investigation is structurally homogeneous along the entire length (Table 1).

To assess the reproducibility of the synthesis method, N_2_ adsorption–desorption isotherms of monoliths from two different synthesis batches have been determined (Figure S2 from Supplementary Materials). The monoliths considered have similar textural properties, in terms of specific surface area and pore volume. In particular, Mono-ICE-1st repetition and Mono-ICE-2nd repetition have, on average, a surface area of 889 and 810 m^2^·g^−1^, respectively. By averaging the two values, the Mono-ICE monoliths have approximately a surface area of 850 m^2^·g^−1^, with a total pore volume of 1.2 cm^3^·g^−1^. Such values are quite close to those (700 m^2^·g^−1^, and pore size of 12 nm) reported by Galarneau et al. for the monoliths prepared with similar procedure [4].

### 2.2. Effect of the Water Treatment

The stability in water of the monolith samples (Mono-ICE-Lateral A, Mono-ICE-Lateral B and Mono-ICE-Central C) and the possible modifications on the textural properties after contacting with water were evaluated by N_2_ physisorption analysis conducted at 77 K. To speed up the process, the three samples were soaked in warm water (T = 50 °C) for 36 h [29]. Samples will be hereafter named Mono-ICE-Lateral A-36h, Mono-ICE-Lateral B-36h and Mono-ICE-Central C-36h, respectively.

**Figure 3:**
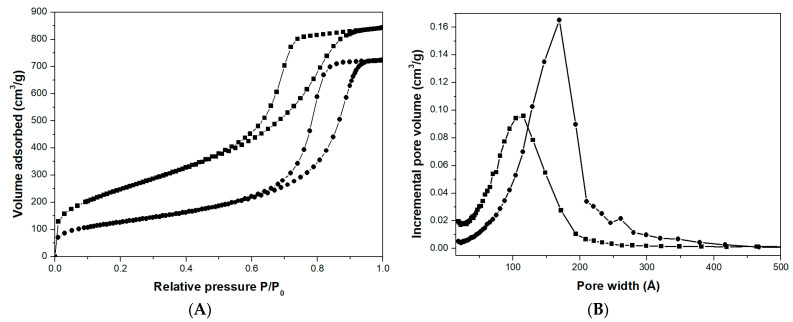
N_2_ adsorption and desorption isotherms at 77 K (**A**) and pores size distribution (**B**) of calcined Mono-ICE-Lateral A (■) and Mono-ICE-Lateral A-36h (●).

For the sake of brevity, only the results about Mono-ICE-Lateral A sample before and after the water treatment are shown in Figure 3. Similar results were obtained for Mono-ICE-Lateral B and Mono-ICE-Lateral C samples (Figure S3 from Supplementary Materials).

The isotherms of the material before and after treatment in water at 50 °C show similar shapes, but at low pressure (P/P_0_ < 0.1) the N_2_ adsorption reduced to around 50% after the treatment in water (Figure 3A) because of the strong reduction in the amount of micropores (Figure 3B). The textural properties of the soaked material are indeed modified: (i) the surface area decreases from 909 to 455 m^2^·g^−1^ (Table 1); (ii) the pore size increases from 115 to 169 Å (Figure 3B); (iii) the total pore volume slightly decreases from 1.3 to 1.1 cm^3^·g^−1^ (Table 1). These changes are probably the effect of the hydrolysis and condensation of those parts of silica surrounding the micropores and characterized by a small radius of curvature, leaving a smoother surface inside the pores and enlarging the pore size [30]. Similar effects were also evidenced for other silica samples [29,31].

### 2.3. Toluene Adsorption from the Gas Phase

Toluene adsorption on silica monoliths was studied from qualitative and quantitative point of view by using FT-IR spectroscopy and volumetric analysis.

**Figure 4:**
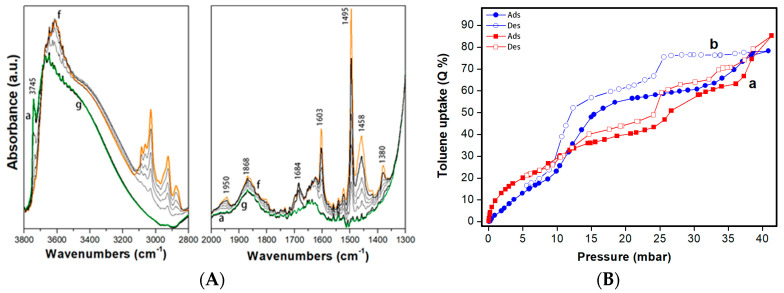
(**A**) FT-IR spectra of calcined Mono-ICE sample after outgassing at RT for 1 h (curve a, green), after dosages of 5, 10, 15, 20 mbar up to 30 mbar of toluene (curve f, orange), and after evacuation of toluene at RT for 30 min (curve g). (**B**) Toluene volumetric adsorption (full symbols) and desorption (empty symbols) isotherms of toluene at 35 °C on calcined Mono-ICE (a, ■), and MCM 41 (b, ●) samples.

The adsorption has been carried out on the Mono-ICE central sample after calcination. The IR spectra obtained after the admission of 30 mbar of toluene on the sample and subsequent gradual decrease of the vapour pressure are reported in Figure 4A.

The admission of increasing toluene pressure on Mono-ICE silica samples (central part) (Figure 4A, curves b–f) results in a progressive disappearance of the band at 3745 cm^−1^, due to isolated Si-OH species, and into the contemporary formation of a broad band centered at 3600 cm^−1^. The progressive disappearance of the band related to isolated silanol species and to the formation of the band at lower frequencies is likely due to O–H···π interactions between silica silanols and toluene molecule [16].

Moreover, bands due to vibrations of the aromatic ring and the methyl group of toluene are also visible at lower wavenumbers. The vibrational modes of toluene adsorbed on the silica surface are described in Table 2.

At higher pressure (above 5 mbar), the adsorption is likely driven by van der Waals interactions between the silica walls and the toluene molecules (host–guest interactions) and among toluene molecules (guest–guest interactions), as already observed elsewhere [16].

After decreasing the toluene pressure and subsequent evacuation at RT, the toluene has been fully desorbed: the band due to isolated SiOH species is completely restored, while the bands of toluene disappear (Figure 4A, curve g).

To gain information on the toluene uptake by the silica monolith, volumetric adsorption measurements at 35 °C were performed (Figure 4B). The isotherm of Mono-ICE sample (curve a) presents a complex shape where three regimes of adsorption are visible. In the adsorption branch, three different plateau are visible between: 5 and 10, 20 and 25, 30 and 35 mbar (with a toluene uptake of ca. 25, 40 and 50%, respectively). This interpretation is confirmed by the almost disappearing signal of the isolated silanol groups at 3745 cm^−1^ in the IR spectrum of Mono-ICE with toluene at 10 mbar (Figure 4A). On increasing the toluene pressure three adsorption steps are found, with a final toluene uptake of 85 Q%, or 1.00 cm^3^·g^−1^ of liquid toluene. The desorption isotherm forms three shallow hysteresis loops in the ranges 10–25, 25–35 and 35–40 mbar are due to a capillary condensation of the toluene molecules inside the pores. This complex behavior could be associated with the filling of the heterogeneous porosity of Mono-ICE sample. Although the calcined monolith showed a monomodal pore size distribution, pores with diameters ranging from 50 to 200 Å are visible, as described before (vide Figure 2).

The maximum toluene uptake is 85 *Q*% at 41 mbar, where

(1) $$Q\%=\frac{m\;adsorbed\;toluene\;\;\left(\mathrm{mg}\right)}{100\;\mathrm{mg}\;of\;sample}\;$$

The volumetric adsorption of toluene on silica monoliths has been compared to that of the reference mesoporous silica MCM-41 in the form of powder (Figure 4B, curve b) and already discussed in our recent publication [16]. For the sake of clarity, textural data of this sample are reported as supporting information (Table S1). Similarly to the Mono-ICE sample, the volumetric isotherm of toluene adsorbed on MCM-41 (Figure 4B, curve b) present three regimes of adsorption, however, only two hysteresis loops in the range 9–25 and 25–40 mbar, due to the toluene capillary condensation inside the pores, can be distinguished. The curve appears rapid until 9 mbar, and then the slope increases up to ca. 15 mbar, when the adsorption curve gradually tends to a first plateau at ca. 25 mbar with an uptake of ca. 59 Q%, which is associated with the filling of the fraction of mesopores with diameter lower than 40 Å (see pore size distribution, Figure S3B from Supplementary Materials). At pressures higher than 25 mbar, the slope increases again and progressively until 45 mbar, where the overall toluene uptake is ca. 78 Q%: the filling of the fraction of mesopores with dimensions from 40 to 80 Å likely occurs at this pressure.

It is here worth noticing that the overall toluene uptake for the two samples is similar, reaching 80–85 Q%, however the monolith adsorbs more at low pressure (<10 mbar). This effect should be correlated to the presence of micropores and narrow mesopores (ca. 20–30 Å) in Mono-ICE.

### 2.4. Rhodamine B Adsorption

**Figure 5:**
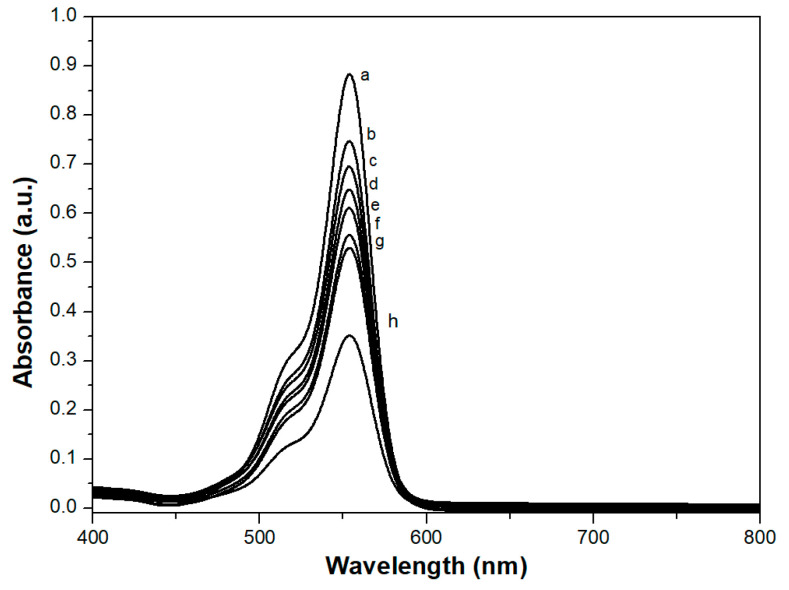
UV-Vis spectra, showing the intensity decrease of the maximum characteristic peak at 553 nm of 1.5 × 10^−2^ mM Rhodamine B in water solution (**a**), after 1 (**b**), 2 (**c**), 3 (**d**), 4 (**e**), 5 (**f**), 6 (**g**) and 24 (**h**) hours of contact with the calcined Mono-ICE, at room temperature.

The adsorption capacity of 1.5 × 10^−2^ mM Rhodamine B by the monoliths was studied by UV-Vis spectroscopy, at room temperature. In particular, the characteristic absorption band at 553 nm of Rhodamine B, due to the π→π* transition of the chromophore unit [32], was observed after contact with the monolith (Figure 5) at increasing times (1 to 24 h).

Figure S6 from Supplementary Materials shows the adsorbed amount of Rhodamine B as a function of concentration of Rhodamine B solution over time.

Rhodamine B is a weak acid (pKa 4.2) with good solubility (34 g·L^−1^) in water. Its organic part is a cation in which the positive charge is shared by the two N atoms. At pH values larger than 4.2, the carboxylic group is predominantly deprotonated and the prevalent form of Rhodamine B is a Zwitterion (Figure S7 from Supplementary Materials). As reported in the literature, Rhodamine B molecules adsorb on the mesoporous silica materials through electrostatic, hydrogen, nonpolar and n–π bonding interaction. The effective adsorption sites on the silica surface are composed mainly of OH and/or oxygen bridges. At a pH between 5 and 6, Zeta potential of silica was negative, and the surfaces of the adsorbent was negatively charged. The positive moieties in the Rhodamine B zwitterion are attracted onto the surface silanolate groups present in silica materials through electrostatic forces. In addition, the residual surface hydroxyls of the mesoporous materials may also form H-bonds with the COO^−^ group present in the Rhodamine B zwitterion [25].

It is reasonable to think that the Mono-ICE monolith, being entirely composed of silica, having a pH of 5.5 and a Zeta potential of −24 mV, behaves exactly in the same way.

**Figure 6:**
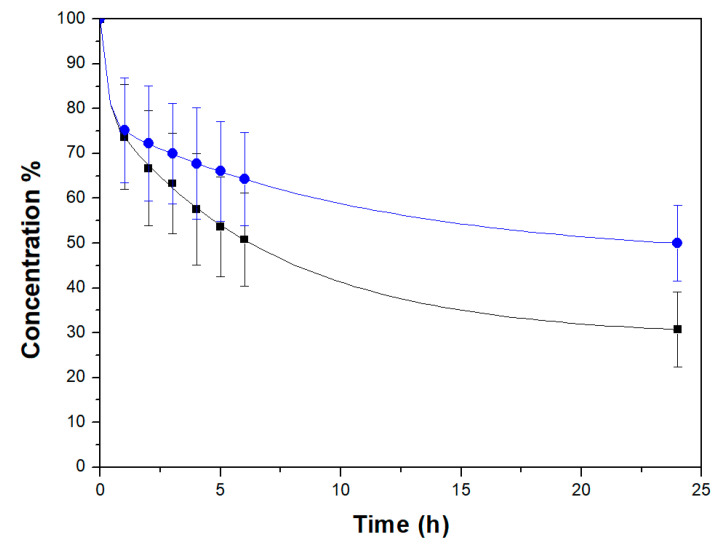
Concentration (%) decrease over time of 1.5 × 10^−2^ mM Rhodamine B water solution in the presence of calcined Mono-ICE before (■) and after water treatment (●). Error bars represent standard deviations calculated on averaging results collected from three replicated experiments.

Figure 6 shows the relative concentration of the Rhodamine B in aqueous solution after contact with Mono-ICE sample at room temperature, before and after it has undergone water treatment, over the time. The measurements have been replicated three times and average values and relative standard deviations are reported in the figure.

The concentration of the Rhodamine B solution decreases over time: 70% of the dye is removed from the solution after 24 h of contact with Mono-ICE before the water treatment (Figure 6, black squares).

The adsorption of Rhodamine B on calcined silica monoliths was compared with that on MCM-41 silica powder, before and after water treatment (Figure S5 from Supplementary Materials). MCM-41 powder (before water treatment) absorbs 58% of Rhodamine B after the first hour of contact, and 92% after 24 h. Rhodamine B adsorbs more rapidly in MCM-41, compared to the Mono-ICE monolith, probably because it is a powder; in the Mono-ICE monolith Rhodamine B has to diffuse in the macropore system before it may reach the mesopores.

After 24 h of contact with Mono-ICE after the water treatment (Figure 6, black circles), the adsorbed amount of Rhodamine B solution decreased by 50%, due to the modification of textural properties (i.e., surface area decrease, the pore size increase, total pore volume decrease) already described (Figure S4B from Supplementary Materials and Table S1).

Similar changes of textural properties are reported for the MCM-41 powder after treatment in water at 50 °C for 36 h (Figure S4 from Supplementary Materials).

MCM-41 silica powder has better performance than the monolith due to the faster diffusion of the pollutant in the pores and having a larger specific surface area; however, the monolith shows excellent adsorption properties and, above all, since it is not synthesized in the form of powder, it is more usable from a technological point of view. As reported in the work of Rasalingam et al. the adsorption capacity of Rhodamine B on MCM-41 silica synthesized by them (having an SSA of 1453 m^2^·g^−1^) is 8.2 × 10^−4^ mol·g^−1^ [25]. The Mono-ICE monolith has an adsorption capacity of 1.07 × 10^−6^ mol·g^−1^. Moreover, unlike MCM-41 silica which is not stable in water, the monolith possesses perfect stability.

MCM-41 is adsorbing more Rhodamine B than Mono-ICE basically for two reasons: (i) the SSA of MCM-41 is larger; (ii) the reduced radius of curvature of the MCM-41 pores favours a stronger interaction with Rhodamine B. In addition, since MCM-41 has a larger tendency to dissolve in water than Mono-ICE because of its thinner walls [16,30], MCM-41 may develop a larger amount of silanol groups per unit surface.

Owing to its instability in water, together with the difficult handing of the powder, the advantages in the use of the MonoICE monolith become evident.

## 3. Materials and Methods

### 3.1. Materials

Silica monoliths were obtained by adapting the procedure described by Galarneau et al. [4]. In detail, a mixture of nitric acid (2.31 g, HNO_3_ 68 % *w*/*w*, Sigma-Aldrich 7697372, M.W. = 63.01 g·mol^−1^), polyethylene oxide-PEO (2.5 g, Sigma-Aldrich 25322683, M.W. > 20 kDa) and deionized water (24.5 g) was prepared and refrigerated at −19 °C for 1 h. Then, tetraethylorthosilicate-TEOS (20 g, Si(OC_2_H_5_)_4_, Sigma-Aldrich 131903, M.W. = 208.33 g·mol^−1^), previously cooled at −19 °C for 1 h, was added to the mixture (molar ratios of the optimized final composition: 1 Si/0.60 PEO/0.26 HNO_3_/14.21 H_2_O).

Polyvinylchloride tubes (five of 8 mm in diameter and seven of 6 mm in diameter and 10 cm length) were then filled with the mixture and closed with a cap, paying particular attention to keep everything cold throughout the whole process by means of an ice bath. Following, the filled tubes were put in a vertical position, held in place by an appropriate support, in a 4 L water bath at 40 °C for 3 days.

**Figure 7:**
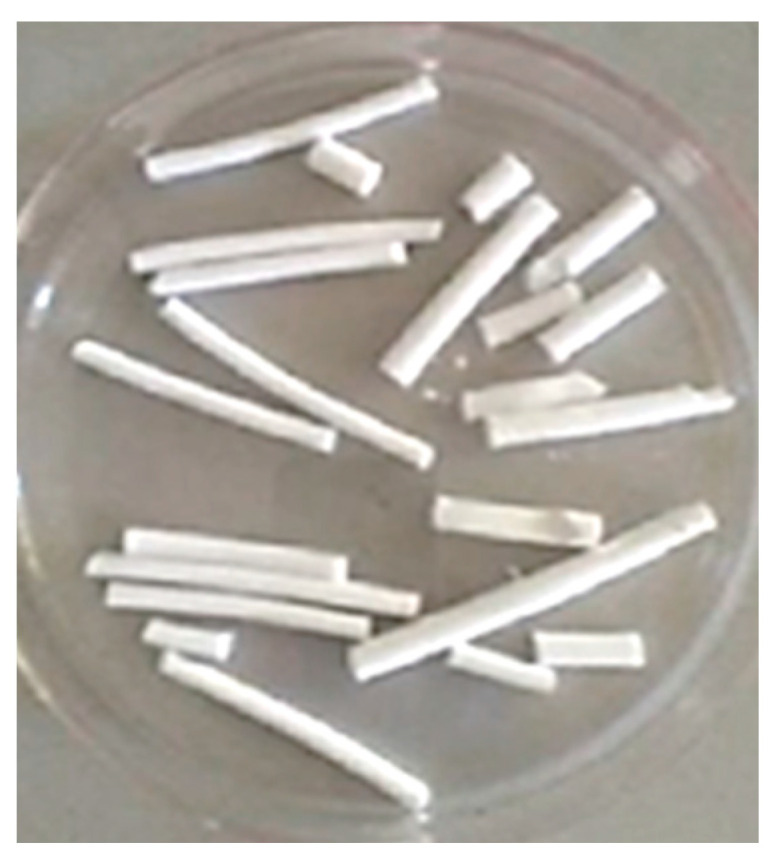
Mono-ICE samples.

The formed monoliths were then removed from the tubes and placed in a water bath at room temperature (RT). The water bath was regenerated every 30 min until neutral pH. The monoliths were then immersed in 1 L of 0.1 M ammonia aqueous solution (NH_4_OH, Sigma-Aldrich 7664417, M.W. = 17.03 g·mol^−1^) in a Teflon bottle, and placed in an oven at 40 °C for 1 day, to catalyze the Ostwald ripening of the weakly condensed silica. Finally, the monoliths were recovered, further washed in a water bath until pH neutrality, dried at RT for 4 days and then calcined in O_2_ flow at 550 °C for 6 h to remove the PEO still present. The obtained silica monoliths were named Mono-ICE (Figure 7).

A monolith was divided into 3 parts of 1 cm in length each, and weighed about 50 mg: 48.9 mg for Mono-ICE-Lateral A, 50.4 mg for Mono-ICE-Lateral B and 55.5 mg for Mono-ICE-Central C.

### 3.2. Stability in Water Treatments

Considering that silica monoliths are soaked in water during their usage as pollutants removers from aqueous phase, the stability in water is as an important characteristic that the adsorbent must have.

To study the stability in water of silica monoliths (and the reference MCM41 silica powder), the solids (50 mg) were dispersed in 5 mL of water and the dispersion was heated at 50 °C for 36 h to speed possible degradation processes. Samples were then recovered and dried at 60 °C for 24 h.

### 3.3. Characterization Techniques

#### 3.3.1. Scanning Electron Microscopy (SEM)

SEM images were acquired on a Quanta 200 scanning electron microscope (FEI, Eindhoven, The Netherlands) equipped with tungsten filament as electron source. Before the analysis, a conductive coating of gold was deposited on the monoliths by low-pressure plasma to avoid that insulating particles are electronically charged under the electron beam.

#### 3.3.2. Infrared Spectra

Infrared spectra were collected by using a Thermo Electron Corporation (Whaltham, MA, USA) FT Nicolet 5700 spectrometer with 4 cm^−1^ resolution. Self-supporting pellets of Mono-ICE samples were obtained by grinding a piece of silica monolith and compressing the obtained powder with a mechanical press at ca. 7 tons·cm^−2^. To perform the analysis, the obtained pellets were placed into an IR cell equipped with KBr windows permanently attached to a vacuum line (residual pressure ≤ 1 × 10^−3^ mbar), allowing all treatments (and toluene adsorption/desorption experiments) to be carried out in situ. All the spectra were collected at beam temperature (ca. 35 °C) on samples previously dehydrated at RT for 30 min to completely remove the adsorbed water.

#### 3.3.3. Nitrogen Adsorption Measurements

Nitrogen Adsorption Measurements were conducted at AlfatestLAB S.R.L (Cinisello Balsamo, Italy). Experiments were performed at 77 K in the pressure range between 0.01 and 1 P/P_0_ of relative pressure using a 3Flex instrument (Micromeritics, Norcross, GA, USA). Prior to adsorption, the samples were outgassed and thermally treated as follows: 3 h at RT, 30 min at 50 °C, 30 min at 80 °C, 2 h at 110 °C and 3 h at 220 °C. The specific surface area of the samples was determined by the Brunauer−Emmett−Teller (BET) multipoint method in the range between 0.05 and 0.25 P/P_0_. The pore size distribution was also calculated by applying the BJH (Barrett, Joyner, and Halenda) method on the adsorption branch (Thickness Curve: Halsey, Correction: Standard).

### 3.4. Toluene Adsorption

Volumetric Analysis. Toluene adsorption isotherms by gas phase were obtained at 35 °C by volumetric analysis of vapor sorption employing an Autosorb-iQ instrument (Quantachrome Instruments, Boynton Beach, FL, USA). Prior to adsorption, the samples were outgassed (final pressure 7 × 10^−4^ mbar) and thermally treated as follows: 30 min at 50 °C, 30 min at 80 °C, 2 h at 120 °C, 2 h at 150 °C and, finally, 12 h at 220 °C in order to remove completely adsorbed water.

### 3.5. Rhodamine B Adsorption

The Rhodamine B adsorption experiments were conducted by using a Lambda 900 UV-Visible spectrometer (Perkin Elmer, Waltham, MA, USA). Rhodamine B (CAS Number: 81-88-9, IUPAC name: [9-(2-carboxy phenyl)-6-diethylamino-3-xanthenylidene]-diethylammonium chloride, was purchased from Sigma Aldrich, St. Louis, MO, USA, (Analytical Standard). Before the experiment, a calibration line was obtained from five Rhodamine B standard solutions (2 × 10^−2^, 5 × 10^−3^, 2.5 × 10^−3^, 1.25 × 10^−3^, and 6 × 10^−4^ mM), resulting in the equation A = 85.702 × C, with *R^2^* = 0.99995.

For the contact tests, 317 mg of sample was placed in a sealable glass vial with 63.4 mL of Rhodamine B solution 1.46 × 10^−2^ mM (pH = 5.5). The bottles have been subsequently sealed with teflon-lined caps and placed on a mechanical stirrer (300 rpm) at RT. At different interval times (i.e., 1, 2, 3, 4, 5, 6, 24 h), an aliquot of each solution (ca. 1 mL) was then withdrawn and directly analyzed. Each experiment was repeated 3 times, every time using a fresh monolith.

Z potential of the monolith at the pH value of the Rhodamine B solution (pH = 5.5) was −24.6 mV.

## 4. Conclusions

In this work a synthesis procedure of mesoporous silica monoliths suitable for being used as adsorbents of organic pollutants in water (Rhodamine B), or in the gaseous phase (toluene, as a model of VOCs), was adopted.

Textural properties were found to be homogeneous over the entire length of the monolith, which has an average surface area of ca. 850 m^2^·g^−1^ and a total pore volume of 1.2 cm^3^·g^−1^. After a water treatment (36 h at 50 °C), the specific surface area remained approximately 500 m^2^·g^−1^, and the total pore volume 1.1 cm^3^·g^−1^.

From a comparison between Mono-ICE and MCM-41 silica powder, it was found that both samples adsorb comparable amounts of toluene, and that the silica monolith performs better at low pressure (<10 mbar).

Monoliths before and after the water treatment were tested as adsorbents for Rodamine B in aqueous solution. Although the water treatment reduced the specific surface area, the treated material was still able to adsorb 50% of the Rhodamine B with respect to 70% of the control sample.

The silica monolith prepared in this study shows slightly worse performances in the adsorption of Rhodamine B when compared to the silica MCM-41 powder, however it displays several advantages including greater ease in handling and recovering.

## Figures and Tables

**Table 1:** Specific Surface Area and Total Pore Volume of Calcined Mono-ICE-Lateral A, Mono-ICE-Lateral B and Mono-ICE-Central C Samples, Before and After 36 h of Water Treatment.

Sample	SSA_BET_^1^ (m^2^·g^−1^)		V_P_^2^ (cm^3^·g^−1^)	
	(Before)	(After)	(Before)	(After)
Mono-ICE Lateral A	909	455	1.3	1.1
Mono-ICE Central C	906	465	1.3	1.1
Mono-ICE Lateral B	853	553	1.2	1.3

^1^ Brunauer-Emmet-Teller (BET) specific surface area (SSA); ^2^ Total pore volume by Barrett, Joyner, and Halenda (BJH) method.

**Table 2:** IR Bands Formed after the Adsorption of Toluene on Mono-ICE.

Frequency (cm^−1^)	Vibration Mode
3085, 3060, 3030	ν C-H aromatic ring
2922, 2871	ν_s_ and ν_as_ C-H toluene methyl group
1603	Quadrant stretching mode of mono-substituted ring C=C bond
1495	Semicircular stretching vibration mono-substituted aromatic ring
1460, 1377	Out-of-phase and in-phase deformations of methyl group

## Data Availability

Not available.

## Supplementary Materials

The following are available online: Figure S1. FT-IR spectra of self-supported pellets of Mono-ICE calcined sample after treatment in vacuum at beam temperature (b.t.) for 30 min; Figure S2. Comparison between the first repetition (Frame A) and the second repetition (Frame B) of N2 adsorption and desorption isotherms of Mono-ICE-A (a), Mono-ICE-B (b) and Mono-ICE-C (c); Figure S3. N2 adsorption and desorption isotherms (Frame A) and pore size distribution (Frame B) of Mono-ICE-A-36h (a), Mono-ICE-B-36h (b) and Mono-ICE-C-36h (c). Figure S4. N_2_ adsorption and desorption isotherms (Frame A) and pores size distribution (Frame B) of MCM-41 -before and after water treatment at 50 °C for 36 h; Figure S5. Concentration (%) decrease over time of 1.5 × 10^−2^ mM Rhodamine Rhodamine B in water solution in the presence of commercial MCM-41 powder. Table S1. Main textural features of MCM-41 silica.

## Sample Availability

Samples of the compounds are available from the authors.
