# Supplementary material for: Silica Monolith for the Removal of Pollutants from Gas and Aqueous Phases
Source: Molecules. 2021 Mar 1;26(5):1316. doi: 10.3390/molecules26051316 (PMC7957575; doi:10.3390/molecules26051316)
Supplement: Supplementary file 1 [file molecules-26-01316-s001.pdf]

molecules

Supplementary Materials

Silica Monolith for the Removal of Pollutants from Gas and Aqueous Phases

Vanessa Miglio [1,2], Chiara Zaccone [1,2], Chiara Vittoni [1,2], Ilaria Braschi [2,3,*], Enrico Buscaroli [3], Giovanni Golemme [4], Leonardo Marchese[1,2] and Chiara Bisio [1,2,5,*]

1 Department of Sciences and Technological Innovation and Interdisciplinary Nano-SiSTeMI Centre, University of Eastern Piedmont A. Avogadro, viale T. Michel 11, 15121 Alessandria, Italy; vanessa.miglio@uniupo.it (V.M.), chiara.zaccone@uniupo.it (C.Z.), chiara.vittoni@uniupo.it (C.V.), leonardo.marchese@uniupo.it (L.M.)
2 Interdisciplinary Nano-SiSTeMI Centre, University of Eastern Piedmont A. Avogadro, viale T. Michel 11, 15121 Alessandria, Italy
3 Department of Agricultural and Food Sciences, University of Bologna, viale G. Fanin 44, 40127 Bologna, Italy; enrico.buscaroli2@unibo.it
4 Department of Environmental Engineering, University of Calabria, Via P. Bucci 45A, 87036 Rende, Italy; giovanni.golemme@unical.it
5 CNR-SCITEC Istituto di Scienze e Tecnologie Chimiche "Giulio Natta", via G. Venezian 21, 20133 Milano, Italy
* Correspondence: chiara.bisio@uniupo.it (C.B.); ilaria.braschi@unibo.it (I.B.); Tel.: +39 0131 360216 (C.B.); +39 051 2096533 (I.B.)

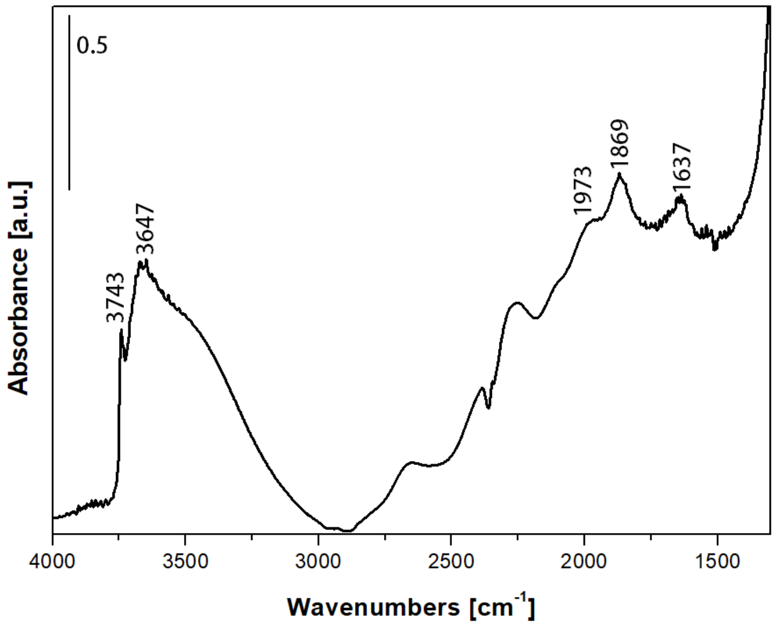

Figure S1. FT-IR spectra of self-supported pellets of Mono-ICE calcined sample after treatment in vacuum at beam temperature (b.t., 35 °C) for 30 min.

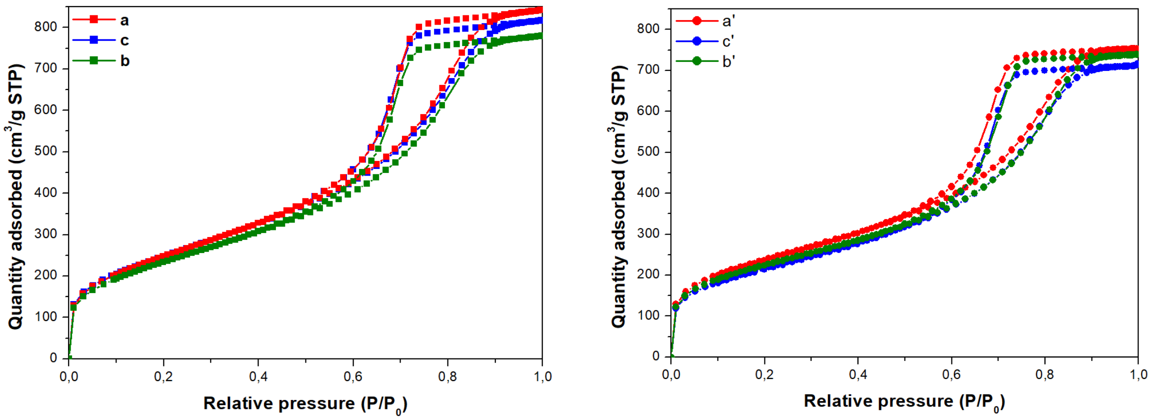

(**A**) (**B**)

Figure S2. Comparison between the first (Frame **A**) and the second repetition (Frame **B**) of N_2 adsorption and desorption isotherms of Mono-ICE-A (**a**), Mono-ICE-B (**b**) and Mono-ICE-C (**c**).

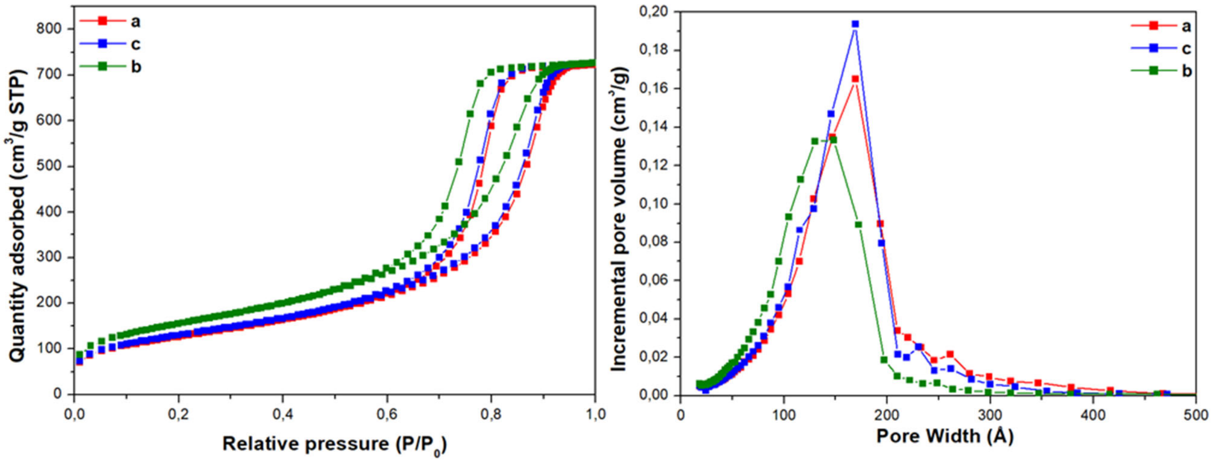

(**A**) (**B**)

Figure S3. N_2 adsorption and desorption isotherms (Frame **A**) and pore size distribution (Frame **B**) of Mono-ICE-A-36h (**a**), Mono-ICE-B-36h (**b**) and Mono-ICE-C-36h (**c**).

Table S1. Main Textural Features of MCM-41 Silica.

Sample	SSA_{BET} [1] [m²/g]	V_T [2] [cm³·g⁻¹]	V_{mesop} [3] [cm³·g⁻¹] 30-80 Å	V_{mesop} [3] [cm³·g⁻¹] 20-100 Å	V_{mesop} [3] [cm³·g⁻¹]	
					20-65 Å	65-100 Å
MCM-41	1103	1.31	0.90	0.95	0.83	0.12

[1] Brunauer-Emmet-Teller (BET) specific surface area (SSA); [2] Total pore volume by NLDFT method; [3] Volume of mesopores NLDFT method.

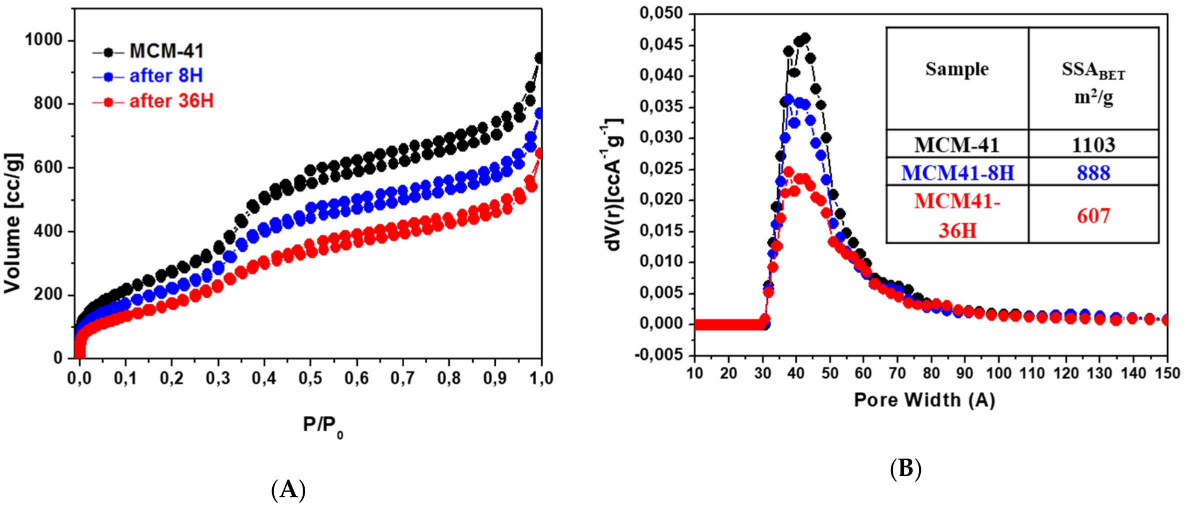

Figure S4. N_2 adsorption and desorption isotherms (Frame **A**) and pore size distribution (Frame **B**) of MCM-41 - before and after water treatment at 50 °C for 36 h.

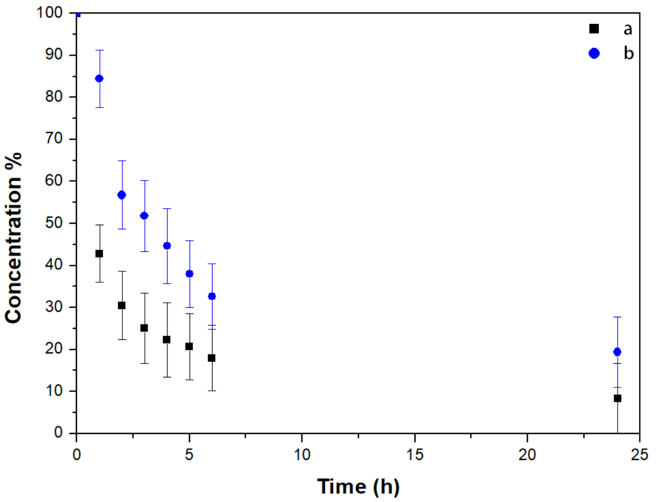

Figure S5. Relative concentration (%) over time of 1.5×10^{-2} mM Rhodamine B in water solution in the presence of commercial MCM-41 powder, before (a■) and after (b●) water treatment.

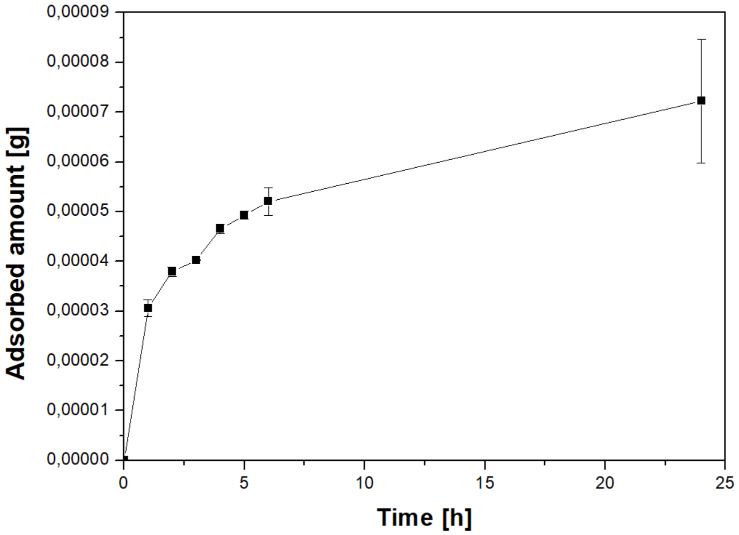

Figure S6. Adsorbed amount (g) over time of 1.46 × 10^{-2} mM Rhodamine B in water solution (63.4 mL) in the presence of Mono-ICE (317 mg).

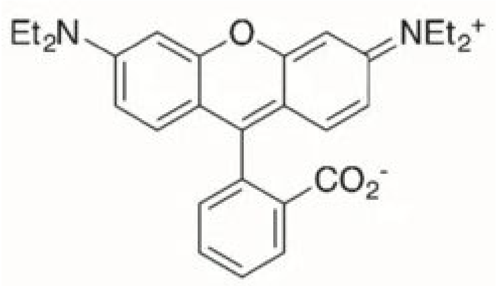

Figure S7. Zwitterionic form of Rhodamine B prevailing in water at pH ≥ 4.2
